# *Bacillus* spp. Potentiate the Virulence and Intracellular Invasion of *A. paragallinarum* in Chickens

**DOI:** 10.3390/ani15142076

**Published:** 2025-07-14

**Authors:** Jiajia Zhu, Ying Liu, Ting Gao, Yunsheng Chen, Keli Yang, Wei Liu, Kui Zhu, Danna Zhou

**Affiliations:** 1 Institute of Animal Husbandry and Veterinary Medicine, Hubei Academy of Agricultural Sciences, Wuhan 430064, China; xmszjj@hbaas.ac.cn (J.Z.);; 2 Institute of Animal Husbandry and Veterinary Medicine, Beijing Academy of Agriculture and Forestry Sciences, Beijing 100097, China; liuyingcau@sina.com; 3 College of Veterinary Medicine, China Agricultural University, Beijing 100193, China

**Keywords:** *Bacillus* spp., *A. paragallinarum*, coinfection, bacterial interaction, NAD, antibiotic resistance

## Abstract

Coinfection poses severe threats to poultry health, particularly due to the complexity and resilience of multispecies interactions, increasing the difficulty of treatment. *Haemophilus* spp., a heterotrophic bacterium, heavily relies on extracellular growth factors acquired from other organisms or its surrounding environment. Although coinfections by *Avibacterium paragallinarum* and *Bacillus* have been reported, the underlying mechanism of the cooperative interaction remains poorly understood. In this study, we characterized the growth-promoting properties and nicotinamide adenine dinucleotide production of some *Bacillus* species, including probiotic *Bacillus*, to evaluate the feasibility of *A. paragallinarum* coinfection in vitro. Meanwhile, we determined the minimum inhibitory concentration (MIC) and population dynamics of cocultured *Bacillus* and *A. paragallinarum* to assess the effect of bacterial interactions on antibiotic efficacy. Additionally, we demonstrated that *B. cereus* aggravates rhinitis symptoms in chickens infected with *A. paragallinarum*. Our findings reveal that *Bacillus* spp.-derived metabolites sustain *A. paragallinarum* growth and enhance its survival, thereby highlighting the infection risks associated with *Bacillus* colonization in the respiratory tract.

## Simple Summary

Bacterial coinfection poses severe threats to poultry health. One common bacterium, *Avibacterium paragallinarum*, relies on extracellular growth factors acquired from other organisms or its surrounding environment and is more susceptible to coinfection. In this study, *Bacillus* promotes the growth and infection of *Avibacterium paragallinarum* in vitro and in vivo by metabolites, especially some *Bacillus* strains isolated from probiotics. This study highlights the necessity for enhanced safety assessments of probiotic *Bacillus* strains to evaluate their potential role in facilitating coinfections.

## 1. Introduction

Coinfections frequently involve synergistic interactions among pathogens, commensals, or opportunistic bacteria through cross-feeding and cross-protection mechanisms [1]. In multispecies communities, complex interactions facilitate metabolite sharing, which diminishes antibiotic efficacy against co-inhabiting microbes [2,3,4]. Metabolic exchanges shape the structure and function of microbial communities, playing a crucial role in ecological and environmental interactions [5]. For instance, opportunistic *Klebsiella pneumoniae* cross-feeds *Acinetobacter baumannii* with sugar fermentation by-products, enhancing the production of dual-species biofilms that confer resistance to antibiotic treatments [6,7]. Such metabolite exchange among community strains can enhance bacterial survival or virulence in resource-limited environments [8,9,10]. These cooperative behaviors among bacteria exacerbate the progression of bacterial infections, leading to more severe diseases and increasing the challenges of antibiotic treatment.

The respiratory tract harbors trillions of bacteria, serving as the second-largest microbial reservoir in the host and acting as gatekeepers of respiratory health [11]. Infectious coryza (IC), an acute respiratory infection in poultry, is caused by *Avibacterium paragallinarum*—a nutrient-deficient pathogen reliant on environmental nicotinamide adenine dinucleotide (NAD^+^) for growth [12]. While vaccination remains the most effective control strategy, antibiotic treatment shows limited efficacy. Previous studies demonstrate that the resident bacteria *Staphylococcus chromogenes* provides the NAD^+^ and releases the NAD^+^ from host cells to promote the survival and growth of *A. paragallinarum* [13]. Furthermore, a positive correlation between *Bacillus* spp. isolates and *A. paragallinarum* has been observed [14], suggesting *Bacillus* spp. may facilitate *A. paragallinarum* infection in the chicken respiratory tract. This potential role is particularly relevant given that certain *Bacillus* species are promising antibiotic alternatives in the feed industry due to their ability to modulate microbial communities [15]. However, some *Bacillus* strains isolated from probiotic products have been associated with sepsis, intestinal inflammation, and liver damage in various mouse models [16]. Given the limited research on *Bacillus* in the respiratory tract, it remains unknown whether and how *Bacillus* spp. assist the survival and infection of *A. paragallinarum* in vivo.

In this study, we investigated the in vitro growth-promoting abilities of *Bacillus* spp.—including probiotic *Bacillus*—on *A. paragallinarum* and elucidated the underlying mechanisms. We observed a significant increase in bacterial numbers when cocultured *Bacillus* spp. and *A. paragallinarum*, this function was attributed to *Bacillus*-derived NAD^+^ production. Furthermore, *B. cereus* CAU492 and *B. licheniformis* YC3-2 provided antibiotic protection against *A. paragallinarum*, as evidenced by an increased minimum inhibitory concentration in the coculture. Additionally, the presence of *Bacillus cereus* CAU492 significantly aggravated clinical symptoms and increased the burden of *A. paragallinarum* in chickens. Our findings indicate that *B. cereus* and *B. licheniformis* present in the respiratory tract promote the replication and infection of *A. paragallinarum* through NAD^+^-feeding and antibiotic protection mechanisms.

## 2. Materials and Methods

### 2.1. Coculture of Various Bacillus Strains and A. paragallinarum

*Bacillus* [16] and *A. paragallinarum* [13] strains were used in this study, previously isolated from various sources, including IC chickens, pig farms, meat products, and probiotic products (Table S1). Overnight cultures of *Bacillus* and *A. paragallinarum* were adjusted to a McFarland turbidity (McF) of 0.5. The bacteria density of *A. paragallinarum* was then diluted to 10^6^ CFU/mL. Next, 100 µL of the bacterial suspensions were spread onto Tryptic Soy Agar (TSA, Land Bridge Technology, Beijing, China) plates supplemented with 5% sheep blood. After air-drying, 5 µL aliquots of *Bacillus* cultures were spotted onto the center of each TSA agar plate. The plates were incubated at 37 °C under 5% CO_2_ for 24 h. After incubation, the growth radii of both the central *Bacillus* colonies and the surrounding *A. paragallinarum* were measured accurately.

### 2.2. Hemolytic Activity Assessment of Bacillus Strains

The hemolytic activity assessment method referenced established protocols [17]. Briefly, *Bacillus* strains were grown overnight in Tryptic Soy Broth (TSB, Land Bridge Technology, Beijing, China) at 37 °C with shaking at 200 rpm and adjusted to 0.5 McF. Then, 5 µL of each *Bacillus* strain was carefully spotted onto the center of TSA agar plates supplemented with 5% sheep blood. The plates were incubated at 37 °C for 24 h, after which the radius of the hemolytic zone surrounding each *Bacillus* colony was measured at the specified time points.

### 2.3. Coculture of Bacillus Strains and A. paragallinarum In Vitro

The murine alveolar macrophage cell line MH-S (ATCC CRL-2019) was used to assess the cellular entry capacity of *A. paragallinarum*. Cells were seeded in 24-well plates at 0.5 × 10^6^ cells per well and cultured overnight at 37 °C under 5% CO_2_. Overnight cultures of the *A. paragallinarum* strains X1-1S-1, *B. cereus* CAU 492, and *B. licheniformis* YC 3-2 were washed once with sterile phosphate-buffered saline (PBS) and resuspended in basal cell culture medium to a final concentration of 2 × 10^6^ CFU/mL. Prior to infection, the MH-S cells were washed with PBS twice. For single infections (MOI = 1), the wells received 250 μL of *A. paragallinarum* suspension plus 250 μL culture medium to achieve a multiplicity of infection. In the coinfection group, 250 μL of each bacterial suspension was added to achieve an MOI of 1 for both strains. The cells were incubated for 6 h at 37 °C in a CO_2_ incubator. After incubation, the culture medium was collected for serial dilution plating. The cells were washed twice with PBS, lysed with 0.1% Triton X-100 (Beyotime Biotechnology Co., Shanghai, China), and the cell lysate was plated on TSA-Van plus agar plates to enumerate the *A. paragallinarum* and TSA agar plates for *Bacillus* quantification.

### 2.4. NAD Measurement

Overnight cultures of *Bacillus* strains were adjusted at 0.5 McF followed by a 1:100 dilution in fresh TSB for 8 h shaking at 37 °C and 200 rpm. After incubation, the fermented supernatants of these *Bacillus* cultures were obtained by centrifugation at 8000× *g* for 5 min at 4 °C. The concentrations of extracellular total NAD (both NAD^+^ and NADH) levels in the *Bacillus* supernatants were measured using an NAD^+^/NADH Assay Kit with WST-8 (Beyotime Biotechnology Co., Shanghai, China) according to the manufacturer’s instructions.

### 2.5. Antibiotic Susceptibility Testing

The minimum inhibitory concentration (MIC) of antibiotics against *A. paragallinarum* and *Bacillus* strains was determined using the micro-broth method as described by the Clinical and Laboratory Standards Institute (CLSI) 2023 guidelines [18]. *Bacillus* and *A. paragallinarum* strains were cultured overnight at 37 °C with shaking at 200 rpm and adjusted to 0.5 McF in cation-adjusted Mueller–Hinton broth (CMHB, GuanDao Biotech Co., Shanghai, China) containing 5% serum and 20 µg/mL of NAD^+^. Cefotamine, Ofloxacin, Doxycycline, Gentamicin, and Ampicillin were diluted twofold in CAMHB containing 20 µg/mL NAD^+^ and 1% (*v*/*v*) sterile-filtered heat-inactivated chicken serum (Solarbio Life Science Co., Beijing, China). Next, 100 µL of bacterial suspension (5 × 10^5^ CFU/mL in CAMHB plus broth) was mixed with an equal volume of the diluted antibiotic and incubated for 16–18 h at 37 °C in a 5% CO_2_ atmosphere. *Escherichia coli* American Type Culture Collection (ATCC) 25922 and *Staphylococcus aureus* ATCC 29213 served as the quality control strains. Following incubation, the bacterial number of *A. paragallinarum* was counted on TSA plates containing 10 µg/mL of vancomycin, 5% fetal bovine serum (FBS), and 20 µg/mL NAD^+^. The population of *Bacillus* was measured using TSA plates.

### 2.6. DNA Sequencing and Genome Analysis

The genomic DNA of *A. paragallinarum* was extracted using the TIANamp Bacterial DNA Kit (Tiangen Biotech, Beijing, China). The DNA was then fragmented to prepare the library and subjected to sequencing using Illumina NovaSeq 6000 (Illumina, San Diego, CA, USA) in a paired-end model. The virulence factors and antimicrobial resistance genes of *A. paragallinarum* were identified based on VFDB, ResFinder, and the Comprehensive Antibiotic Resistance (CARD) database using BLASTn -2.10.0+ with a cut-off of 60% of coverage and 80% of identity. The whole-genome sequences of *B. cereus* CAU492 and *B. licheniformis* YC3-2 were deposited at GenBank under accession numbers PRJNA1134002 and PRJNA1133239. The *A. paragallinarum* used in this study had been submitted before under accession number PRJNA648655.

### 2.7. Infection Challenge Experiments in Birds

For the animal experiments, six-week-old specific pathogen-free (SPF) chickens were purchased from Boehringer Ingelheim Vital Biotechnology Co., Ltd. (Beijing, China) The project strictly adhered to ethical guidelines for laboratory animals and implemented the principles of the 3Rs (Replacement, Reduction, and Refinement) to ensure the appropriate handling of the animals and the collection of animal tissue samples for the construction of a chicken infectious rhinitis animal infection model.

The bird infection test followed the procedures in a previous study [13]. The chickens were randomly assigned to six groups, control, *B. cereus* CAU492 only, *B. licheniformis* YC3-2 only, *A. paragallinarum* only, *B. cereus* CAU492 coinfected with *A. paragallinarum*, and *B. licheniformis* YC3-2 coinfected with *A. paragallinarum*, with five chickens in each group. The chickens were allowed to acclimate to the environment for one week prior to infection, following standardized feeding protocols. Two strains of *Bacillus* spp. (*B. cereus* CAU492 or *B. licheniformis* YC3-2) and *A. paragallinarum* 1X-1S-1 were all cultured overnight. After incubation, bacterial density was determined; the bacteria were centrifuged and resuspended using sterile phosphate-buffered saline (PBS) to achieve a final concentration of 5 × 10^8^ CFU/mL. The chickens were pretreated with a bacteria-free solution containing 32 µg/mL of vancomycin by injection in the infraorbital sinuses before infection. Subsequently, the chickens were injected with *Bacillus* spp., followed by infection with *A. paragallinarum* the next day. On the final day of the experiment, the clinical symptoms of chicken sinusitis were assessed and scored [19] based on the following scale: 0 for no clinical signs; 1 for mild signs (slight facial swelling); 2 for moderate signs (facial swelling and nasal discharge); 3 for severe signs (facial swelling, nasal discharge, ocular discharge, half-closed eyes). All the chickens were humanely euthanized via the inhalation of carbon dioxide gas, and nasal and sinus tissues were collected for bacterial counting. Some tissues were fixed with 4% paraformaldehyde for hematoxylin and eosin (H&E) staining. These experiments were performed as three independent experiments at different intervals.

### 2.8. Statistical Analysis

Statistical analysis of the data was performed using GraphPad Prism 9 software. *p*-values were calculated using an unpaired *t*-test with Bonferroni correction between two groups or a nonparametric one-way ANOVA among multiple groups. Linear modeling (LM) was performed in the R package (version 4.1.3).

## 3. Results

### 3.1. Bacillus Facilitates the Growth of A. paragallinarum Under NAD-Deficiency Conditions

**Figure 1:**
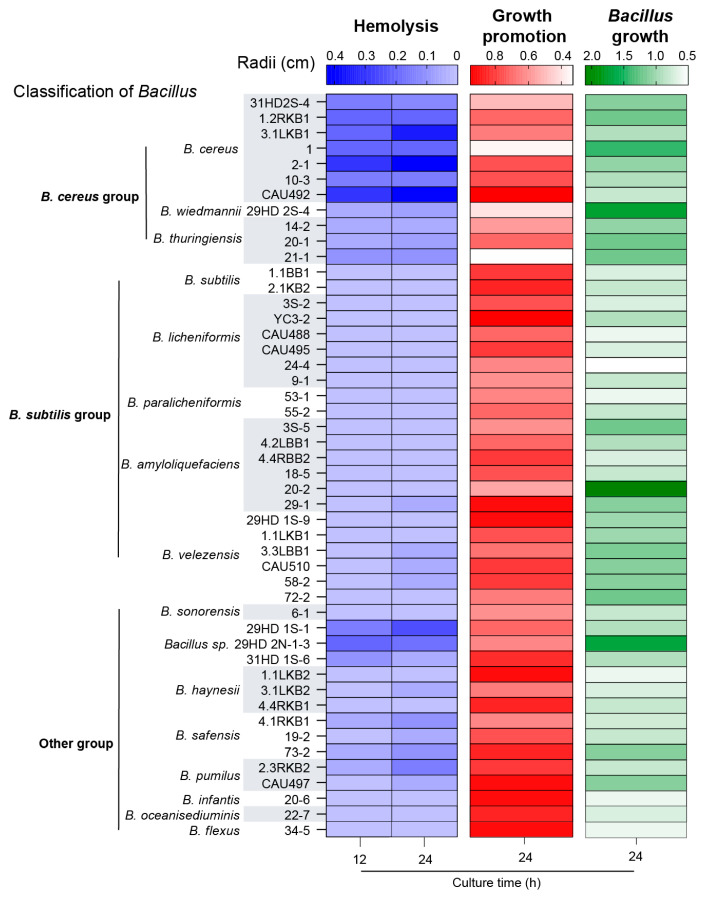
Commensal *Bacillus* promoting *A. paragallinarum* growth. The differences in positive interaction between various genera of *Bacillus* spp. and *A. paragallinarum*. *Bacillus* was selected by the hemolysis and growth-promotion ability. The radii of hemolysis, growth-promotion, and plaque were measured when mono- or cocultured with *A. paragallinarum* on sheep blood plate. Hemolytic activity was color-coded in blue, growth promotion in red, and Bacillus plaque formation in green.

NAD is an essential factor for the growth and survival of *A. paragallinarum*, which is a pathogen known for growth-factor deficiency. Previous studies have confirmed that certain *Bacillus* species can enhance the survival of *A. paragallinarum* in vitro [14]. However, the prevalence of this trait across *Bacillus* species and the comparative efficacy of different strains remain unclear. To address this gap, we conducted in vitro assays to assess whether distinct *Bacillus* strains potentiate *A. paragallinarum* growth and to elucidate their mechanistic role in infection pathogenesis. A total of 51 *Bacillus* strains (representing 17 species) were included in the study. Growth promotion was quantified via the radius of *A. paragallinarum* growth, which ranged from 0.3 to 1.0 cm (Figure S1a). Comparative analysis revealed significant inter-strain variability in growth-promoting activity, with 24 *Bacillus* strains promoting growth radii > 0.7 cm (Figure 1). Additionally, we also measured the microcolony radii of *Bacillus* strains, revealing that species in the *B. cereus* group formed relatively larger bacterial colonies than other *Bacillus* groups (Figure S1b). This prompted investigation of a potential correlation between the *Bacillus* colony size and growth-promoting capacity. Linear regression analysis demonstrated a significant negative correlation (Pearson’s r = −0.51, *p* < 0.05; Figure S1c), indicating an inverse relationship between the *Bacillus* colony dimensions and *A. paragallinarum* growth enhancement.

Given that host cells serve as a substantial nutrient pool for pathogen infection, we further evaluated the hemolysis activity of the *Bacillus* species. All the species in the *B. cereus* group exhibited hemolysis zones, indicating robust hemolytic capacity. In contrast, some species in the *B. subtilis* group (*B. amyloliquefaciens* and *B. velezensis*) displayed only minimal hemolytic activity. Some strains exhibited comparable high levels of hemolysis with *B. cereus* (Figure S1d), like *Bacillus* sp. 29HD 1S-1. Combined growth-promoting and hemolytic abilities, we screened seven strains of *Bacillus* (*B. cereus* 2-1, *B. cereus* 10-3, *B. cereus* CAU492, *B. licheniformis* YC3-2, *Bacillus* sp. 29HD 1S-1, *Bacillus* sp. 31HD 1S-6, *B. pumilus* CAU497, *B. oceanisediuminis* 22-7) for the next experiments. Considering the growth-promoting ability of *Bacillus* strains and their hemolytic activity, we supposed that *B. cereus* is more likely to promote the invasion of *A. paragallinarum* compared to other *Bacillus* species.

### 3.2. NAD Derived from Bacillus Promotes the Growth of A. paragallinarum

**Figure 2:**
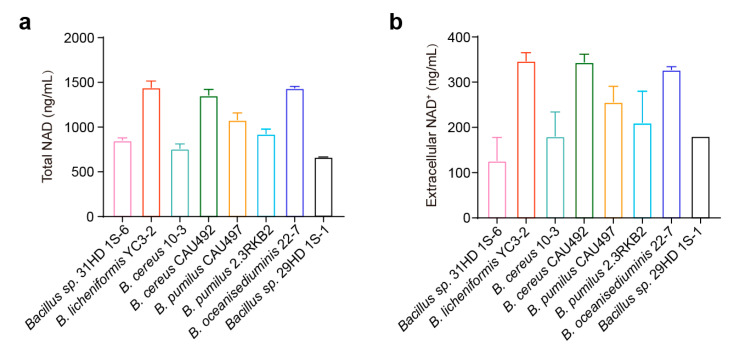
*Bacillus* species release NAD into extracellular. Extracellular concentrations of total NAD (including NAD^+^ and NADH) (**a**) and NAD^+^ (**b**) in *Bacillus* supernatant after 8 h of culturing. The mean of three biological replicates is shown, and error bars represent the standard deviation (SD) (*n* = 3).

Given that *Bacillus* spp. promotes the growth of *A. paragallinarum*, we speculated that *Bacillus* produces some kinds of diffusible metabolic cofactors required by *A. paragallinarum*. To investigate this mechanism, we further measured the NAD content in the culture supernatants, as it has been previously reported that various *Bacillus* species can cause the leakage of NAD. Our findings revealed that the supernatants of the different *Bacillus* species exhibited varying concentrations of total NAD (both NAD^+^ and NADH) (Figure 2a). Notably, *B. licheniformis* YC3-2, *B. cereus* CAU492 and *B. oceanisediuminis* 22-7 exhibited significantly higher total NAD levels than other strains. These results confirm that *Bacillus* species can release NAD extracellularly. Interestingly, these strains also exhibited elevated NAD^+^ production (Figure 2b), with *B. licheniformis* YC3-2 displaying particularly highest levels. The NAD production profile aligned with different growth-promotion ability of *Bacillus* strains, suggesting that *Bacillus*-derived NAD likely sustains the survival of *A. paragallinarum* in chickens with infectious coryza.

### 3.3. Bacillus Enhances the Invasion of A. paragallinarum In Vitro

**Figure 3:**
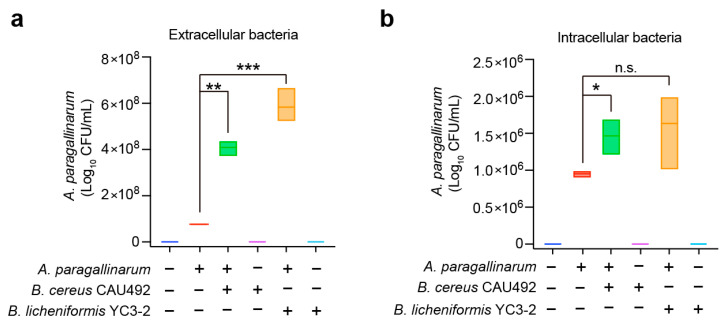
*Bacillus* enhances the cellular invasion capacity of *A. paragallinarum*. The extracellular (**a**) and intracellular (**b**) counts of *A. paragallinarum* were quantified during mono-infection or coinfection with *Bacillus*. Boxplots represent the distribution of *A. paragallinarum* (CFUs, *n* ≥ 3), * *p* < 0.05, ** *p* < 0.01, *** *p* < 0.001, n.s. means no significance.

To investigate whether *Bacillus* promotes the invasion of *A. paragallinarum* in cells, we conducted coculture assays with *B. cereus* CAU492, *B. licheniformis* YC3-2 and *A. paragallinarum* on MH-S cells. *B. cereus* CAU492 was found to increase both extracellular and intracellular bacterial numbers of *A. paragallinarum* (Figure 3a). On the other hand, *B. licheniformis* YC3-2 increased the load of *A. paragallinarum* more significantly in extracellular than intracellular (Figure 3b). Compared to *B. cereus* CAU492, *B. licheniformis* YC3-2 exhibited a significantly greater enhancement in the growth of *A. paragallinarum*. These findings demonstrate that *Bacillus* promotes the invasion and proliferation of *A. paragallinarum* in cells.

### 3.4. Bacillus spp. Confer Protection on A. paragallinarum Against Antibiotics

**Figure 4:**
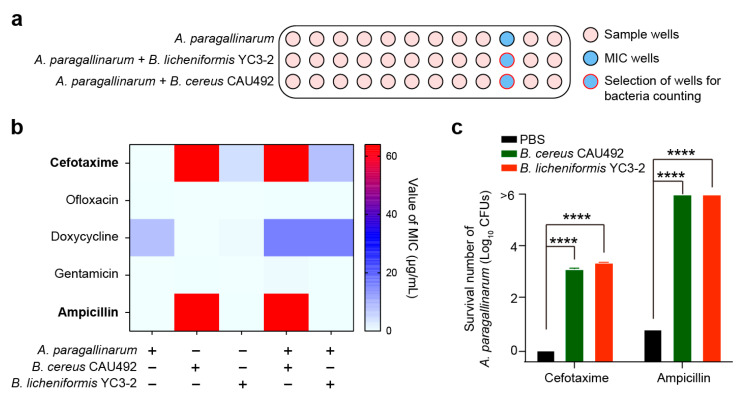
*Bacillus* reduces the antibiotic efficacy against *A. paragallinarum*. (**a**) Diagrams of experimental records. (**b**) The MIC values of *A. paragallinarum* 1X-1S-1, *B. cereus* CAU492, and *B. licheniformis* YC3-2. (**c**) Bacterial numbers of *A. paragallinarum* in the presence of either cefotaxime or ampicillin. **** *p* < 0.0001).

Based on the threat of antimicrobial resistance posed by antibiotics in farms [20], we assessed the growth fitness and antibiotic tolerance of *A. paragallinarum* when cocultured with *Bacillus* in the presence of seven kinds of antibiotics. First, we measured the minimum inhibitory concentration (MIC) of *A. paragallinarum* and *Bacillus* against these antibiotics. Our results showed that *A. paragallinarum* was highly sensitive to Cefotaxime, Ofloxacin, Gentamicin, and Ampicillin. The MIC values of *B. cereus* CAU492 against Ofloxacin and Gentamicin were below 0.25 and 0.5 µg/mL, respectively, while the MICs for the remaining antibiotics were at least 64 µg/mL. *B. licheniformis* YC3-2 displayed resistance to Cefotaxime but increased sensitivity to Ofloxacin, Gentamicin, and Ampicillin (MIC values below 0.125 and 0.25 µg/mL, respectively; Table S2). According to the diagrams, we recorded the MICs of mono-culture and coculture wells and selected wells for bacteria counting (Figure 4a). In the presence of Cefotaxime and Ampicillin, the MIC values of the cocultures were either consistent with or higher than those of *Bacillus* alone (Figure 4b). Further, we counted the bacterial number of *A. paragallinarum* in these groups of wells and found a significant increase-from undetectable (0 CFU) to 1250 and 2250 CFU-following Cefotaxime treatment (Table S3). This represented an increase of more than 1000 times compared to the cocultured group alone (Figure 4c). Similarly, the number of *A. paragallinarum* increased from 6 to more than 10^6^ CFUs in the coculture after the Ampicillin treatment. Interestingly, we simultaneously observed that *A. paragallinarum* increased the MIC value of *B. cereus* CAU492 against Doxycycline (Figure 4b), indicating bidirectional antibiotic tolerance modulation. These results indicate that *Bacillus* significantly enhances *A. paragallinarum* survival under antibiotic pressure. Collectively, our findings suggest that *Bacillus* plays an assistant role in the respiratory tract colonization of *A. paragallinarum* by protecting the pathogens from antibiotic-induced eradication.

To further investigate the underlying mechanism of *Bacillus* in enhancing the antibiotic resistance and infection of *A. paragallinarum*, we analyzed the resistance genes and virulence genes carried by *A. paragallinarum* and *Bacillus* via genomic analysis. The results revealed that *A. paragallinarum* carried the tetracycline resistance gene *tet(B)* and the virulence genes (*lpxC*, *manB*, *yhxB*, *gmhA*, and *lpcA*). *B. cereus* CAU492 harbors the resistance genes *bcII*, *bcI*, *fosB*, and *vanZF* and the virulence genes *cytK*, *bas3190*, *nheC*, *nheA*, and *inhA*. Among them, *B. licheniformis* YC 3-2 carried only the resistance genes *ermD*, *bcrA*, *bcrB*, *bcrC* (Table S4). Notably, genomic analysis suggested the potential involvement of β-lactamase genes (*bcII* and *bcI*) in the ability of *B. cereus* CAU492 to hydrolyze cephalosporin antibiotics, contributing to the survival of *A. paragallinarum* in the presence of Cefotaxime and Ampicillin by reducing antibiotic concentration in the environment. Additionally, *B. cereus* CAU492 carries the biosynthesis gene of the hemolytic enterotoxin (*nhe*) that could exacerbate *A. paragallinarum* infections by producing enterotoxin. However, *B. licheniformis* YC 3-2 lacks the β-lactamase gene and still enhances the survival of *A. paragallinarum* in cefotaxime treatment, possibly mediated by other mutualistic mechanisms.

### 3.5. Bacillus Aggravates the Disease Severity in A. paragallinarum-Infected Chickens

**Figure 5:**
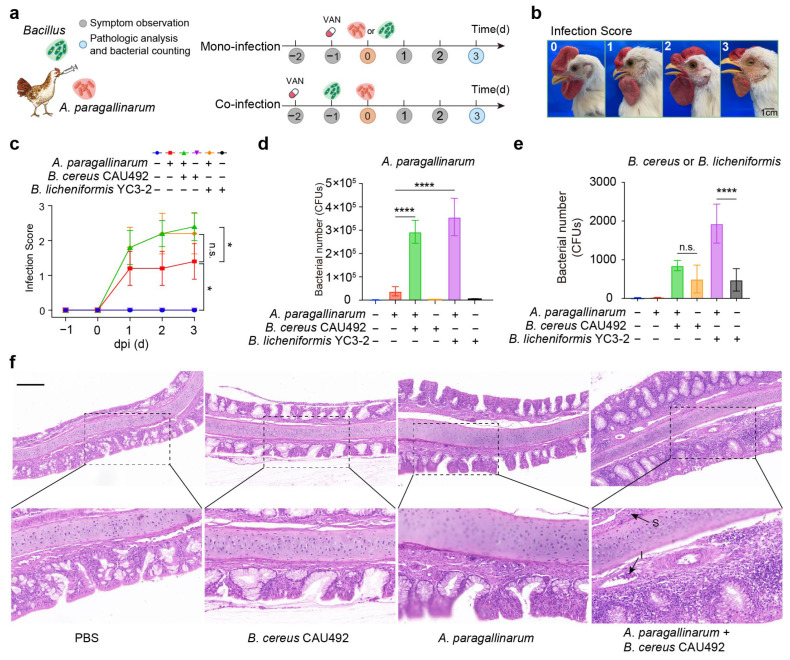
*Bacillus* aggravates *A. paragallinarum*-induced infection in chickens. (**a**) Schematic illustration of the experimental workflow. (**b**) An infection score was used to determine the severity of symptoms, including the swelling of the infraorbital sinus, decreased food intake, and loss of body weight. (**c**) *Bacillus* aggravates the symptoms induced by *A. paragallinarum*. (**d**,**e**) Bacterial burdens of *A. paragallinarum* (**d**), *B. cereus* CAU492 or *B. licheniformis* YC3-2 (**e**) in the nasal cavity and infraorbital sinuses of poultry. (**f**) Tissue damage in the nasal cavity of chicken coinfected with *B. cereus* CAU492 and *A. paragallinarum.* Arrows indicated thickening of the submucosa (S) and infiltration of inflammatory cells (I). Scale bar = 200 µm. *p* values were calculated using an unpaired *t*-test, * *p* < 0.05, **** *p* < 0.0001), n.s. means no significance. The mean of three biological replicates is shown, and error bars represent the standard deviation (SD) (*n* = 5).

Given that *Bacillus* supported the growth and invasion of *A. paragallinarum* in vitro, we subsequently conducted in vivo experiment to investigate whether their positive interactions enhance infection severity. According to the schematic diagram (Figure 5a), six-week-old SPF chickens were randomly divided into six groups (n = 5 per group) and infected with *A. paragallinarum* in the presence or absence of *Bacillus*. Clinical symptoms were carefully evaluated using a 0–3 severity (Figure 5b), reflecting the intensity of nasal inflammation. Our results revealed that *A. paragallinarum* coinfected with *Bacillus* caused more severe clinical symptoms of nasal inflammation in chickens than the mono-infection (Figure 5c). Meanwhile, *B. licheniformis* YC3-2 or *B. cereus* CAU492 caused no visible symptoms of nasal inflammation when infected alone. This finding suggests that *Bacillus* exacerbates *A. paragallinarum*-induced infection in chickens.

To evaluate the cross-feeding dynamics between *Bacillus* and *A. paragallinarum*, we quantified the bacterial load in the nasal cavity and infraorbital sinuses. Our findings illustrated that both *B. licheniformis* YC3-2 and *B. cereus* CAU492 significantly increased *A. paragallinarum* populations (Figure 5d). Similarly, the presence of *A. paragallinarum* increased the population of *B. cereus* CAU492, though the difference was not significant, *B. licheniformis* YC3-2 exhibited a more pronounced increase when coinfected with *A. paragallinarum* (Figure 5e). Notably, histopathological analysis revealed that coinfections induced submucosa thickening, extensive inflammatory cells infiltration, and a disappeared villus structure of epithelial cells (Figure 5f). These results confirm that the introduction of *Bacillus* spp. promote *A. paragallinarum* infection through a combination of metabolic support and protective mechanisms.

## 4. Discussion

The prevailing occurrence of coinfections generally stems from bacteria-positive interactions, including symbiosis and collaboration [21], which are often mediated by various bacterial metabolites produced by each member of the community. These behaviors related to signaling communication among bacteria are ubiquitous and important to ecosystem stability such as exchanging metabolic products and sharing genes [22,23], especially when engaged in auxotrophic and prototrophic bacteria [24]. Such symbiotic interactions may be prevalent in humans and animals, resulting in the exacerbation of coinfections. Some *Bacillus* species are reported to serve as probiotic microbiota engaged in colonization resistance and the antagonism of pathogens by various metabolites [25,26]. While toxin- and mobile antimicrobial-resistant genes also have been found in *Bacillus* probiotics, that pose a risk in application [16]. This study found a coinfection between *Bacillus* and *A. paragallinarum* that revealed a new risk of *Bacillus*. Although probiotic *Bacillus* strains have exhibited in vitro growth-promoting effects on *A. paragallinarum*, their oral administration typically restricts interaction with respiratory pathogens. This limitation may diminish their potential to exacerbate infections relative to *Bacillus* strains from other sources. Nevertheless, the potential risk of *Bacillus*-mediated enhancement of *A. paragallinarum* infections warrants continued attention.

Respiratory infections stand as a prevalent contributor to heightened mortality rates in poultry across the globe [27]. Whereas the bulk of coinfection research has concentrated on viral coinfections [28] and viral–bacterial coinfections [29], investigations into respiratory bacterial coinfections have been comparatively limited. Bacterial cooperation and collaboration often emerge as seemingly “costless” behaviors among bacteria within a community [22]. *A. paragallinarum*, an opportunistic pathogen auxotrophic for NAD, relies on the support of host cells or bacteria in the surrounding environment for this essential cofactor during survival and infection [30]. Based on its specificity, research has identified instances of coinfection between *A. paragallinarum* and other bacteria, such as *A. paragallinarum* and *Gallibacterium anatis* being coinfected in chickens, causing the aggravation of infectious coryza [31]. These coinfection cases reveal mutualistic behavior by metabolite sharing that is vital for the survival of pathogen persistence. Our findings demonstrate that various *Bacillus* species exhibit distinct growth-promoting behaviors on *A. paragallinarum* through abundant NAD^+^ synthesized by *Bacillus* species, as bacterial NAD^+^ serves as an essential redox factor in bacterial energy generation and the synthesis of metabolic products [32,33]. Extracellular NAD^+^ was detected in the *Bacillus* cultures, indicating that *Bacillus* can synthesize its own energy and excrete excess NAD^+^ into the environment. Based on the importance of NAD^+^ in regulating cellular metabolism and proliferation [34,35], the behavior of the NAD release process may be involved in specific metabolic pathways in *Bacillus*, such as its biofilm formation [36]. However, whether the secretion behavior is active or passive and the underlying regulatory mechanism remain to be further investigated. Furthermore, the hemolytic properties of *Bacillus* represent virulence factors that damage host cells and may contribute to the exacerbation of *A. paragallinarum* infections.

Compared to *B. licheniformis* YC3-2 in our work, *B. cereus* CAU492 carries multiple enterotoxin genes, including *nheA* and *nheC*, potentially leading to more serious infections in cellular models. Research has demonstrated that some *B. cereus* strains are capable of producing a heat-stable emetic toxin called cereulide and carry mobile antimicrobial resistance genes, posing a significant risk to both humans and animals [16,37]. Therefore, the development of *Bacillus cereus* as a probiotic requires rigorous safety evaluation for its potential to cause infection and coinfection. Crucially, the growth-promoting behaviors exhibited by *Bacillus* species imply that synergistic *Bacillus* colonizing the respiratory tract may serve an assisting role in the colonization and infection processes of *A. paragallinarum*. Interestingly, alongside the increased bacterial population of *A. paragallinarum* while in the coculture, there is also a notable augmentation in the population of *B. licheniformis* YC3-2 (Figure 5e), indicating that the presence of *A. paragallinarum* may facilitate the reproduction of *Bacillus* species in vivo.

Antibiotic resistance is frequently mediated by bacterial metabolites through siderophore production, antibiotic hydrolysis, and biofilm formation [38,39]. For instance, *Pseudomonas aeruginosa* produces pyoverdine and enterobacteria siderophore enterobactin to displace the iron hijack transporters of antibiotic cefiderocol for the cross-protection of susceptible *P. aeruginosa* [40]. Similarly, *Enterococcus* and *Pseudomonas* augment bacterial resistance to antibiotics through biofilm protection [41]. Our study predicted β-lactamase in *B. cereus* CAU492, an enzyme capable of hydrolyzing β-lactam antibiotics [42], may contribute to enhancing *A. paragallinarum* survival during cephalosporin or ampicillin exposure. This phenomenon is akin to certain strains of *E. coli* producing β-lactamase, which enables *Salmonella* Typhimurium to evade antibiotic eradication [43]. The antibiotic protection of *B. licheniformis* YC3-2 may contribute by biofilm formation [44] or other resistant genes, such as the *bcrA*, *bcrB*, or *bcrC* genes, which mediated Bacitractin resistance [45]. Remarkably, the cocultured bacteria displayed heightened resistance to antibiotic doxycycline, likely due to the presence of the tetracycline resistance gene *tet(B)* in *A. paragallinarum*, which confers tetracycline antibiotic resistance [46,47]. Collectively, these mechanisms demonstrate how bacterial metabolite exchange provides cross-protection to members engaged in antibiotic battles within niches.

## 5. Conclusions

To our knowledge, this study represents a novel investigation of the impact of *Bacillus* species on respiratory bacterial infections. Through experimental observations, we have revealed a syntrophic mechanism in which *B. cereus* and *B. licheniformis* promote the virulence of *A. paragallinarum* by supplying essential nutrients (NAD^+^) and antibiotic-resistant substances. These discoveries not only expand our understanding of bacterial coinfection mechanisms but also provide critical guidance for the rational development and clinical application of *Bacillus*-based probiotics.

## Data Availability

The original contributions presented in this study are included in the article/Supplementary Material. Further inquiries can be directed to the corresponding authors.

## Supplementary Materials

The following supporting information can be downloaded at: https://www.mdpi.com/article/10.3390/ani15142076/s1, Figure S1: *Bacillus* promotes *A. paragallinarum* growth; Table S1: Sources of bacteria used in this study; Table S2: Minimum inhibitory concentration for mono-cultures and cocultures (1:1) of *Bacillus* and *A. paragallinarum*; Table S3: Bacterial number of *A. paragallinarum* in mono-culture and coculture; Table S4: The presence of resistance genes and toxin genes in *A. paragallinarum* and *Bacillus* spp.

## Abbreviations

The following abbreviations are used in this manuscript:

CLSI	Clinical and Laboratory Standards Institute
NAD^+^	Nicotinamide adenine dinucleotide
MIC	Minimum inhibitory concentration
McF	McFarland turbidity
MOI	Multiplicity of infection
