# Supplementary material for: Bacillus spp. Potentiate the Virulence and Intracellular Invasion of A. paragallinarum in Chickens
Source: Animals (Basel). 2025 Jul 14;15(14):2076. doi: 10.3390/ani15142076 (PMC12291824; doi:10.3390/ani15142076)
Supplement: Supplementary file 1 [file animals-15-02076-s001.zip › animals-3711132-supplementary.pdf]

# *Bacillus* spp. Potentiate the Virulence and Intracellular Invasion of *A. paragallinarum* in Chickens

**Jiajia Zhu [1,†], Ying Liu [2,†], Ting Gao [1], Yunsheng Chen [3], Keli Yang [1], Wei Liu [1], Kui Zhu [3,*] and Danna Zhou [1,*]**

[1] Institute of Animal Husbandry and Veterinary Medicine, Hubei Academy of Agricultural Sciences, Wuhan 430064, China; xmszjj@hbaas.ac.cn (J.Z.); gaoting2017@hbaas.ac.cn (T.G.); keliy6@hbaas.ac.cn (K.Y.); liuwei@hbaas.ac.cn (W.L.)
[2] Institute of Animal Husbandry and Veterinary Medicine, Beijing Academy of Agriculture and Forestry Sciences, Beijing 100097, China; liuyingcau@sina.com
[3] College of Veterinary Medicine, China Agricultural University, Beijing 100193, China; yunshengchen@cau.edu.cn
* Correspondence: zhuk@cau.edu.cn (K.Z.); zdn66@hbaas.ac.cn (D.Z.); Tel.: +82-010-62731686 (K.Z.); +86-027-87156122 (D.Z.)
† These authors contributed equally to this work.

**Table S1** Sources of bacteria used in this study.

| Isolations | Number | Sources of Samples |
|---|---|---|
| *B. cereus* | 31HD2S-4 | Infectious coryza, Handan |
| | CAU492 | Foods |
| | 1.2RKB1, 3.1LKB1 | Infectious coryza,Hebei |
| | 1, 2-1, 10-3 | Probiotic products |
| *B. wiedmannii* | 29HD 2S-4 | Infectious coryza, Handan |
| *B. thuringiensis* | 14-2, 20-1, 21-1 | Probiotic products |
| *B. subtilis* | 1.1BB1 | Infectious coryza, Hbei |
| | 2.1KB2 | Infectious coryza,Tianjin |
| *B. licheniformis* | 3S-2 | Infectious coryza,Tianjin |
| | YC3-2 | Healthy chick respiratory |
| | CAU488, CAU495 | Food |
| | 24-4, 9-1 | Probiotic products |
| *B. paralicheniformis* | 53-1, 55-2 | Probiotic products |
| *B. amyloliquefaciens* | 3S-5 | Infectious coryza, Tianjin |
| | 4.2LBB1, 4.4RBB2 | Infectious coryza, Hebei |
| | 18-5, 20-2, 29-1 | Probiotic products |
| *B. velezensis* | 29HD 1S-9 | Infectious coryza, Handan |
| | 1.1LKB1, 3.3LBB1 | Infectious coryza, Hebei |
| | CAU510 | Food |
| | 58-2, 72-2 | Probiotic products |
| *B. sonorensis* | 6-1 | Probiotic products |
| *Bacillus sp.* | 29HD 1S-1, 29HD 2N-1-3, 31HD 1S-6 | Infectious coryza, Handan |
| *B. haynesii* | 1.1LKB2, 3.1LKB2, 4.4RKB1 | Infectious coryza, Hebei |
| *B. safensis* | 4.1RKB1 | Infectious coryza, Hebei |
| | 19-2, 73-2 | Probiotic products |
| *B. pumilus* | 3.3RKB2 | Infectious coryza,Hebei |
| | CAU497 | Food |
| *B. infantis* | 20-6 | |
| *B. oceanisediuminis* | 22-7 | Probiotic products |
| *B. flexus* | 34-5 | |

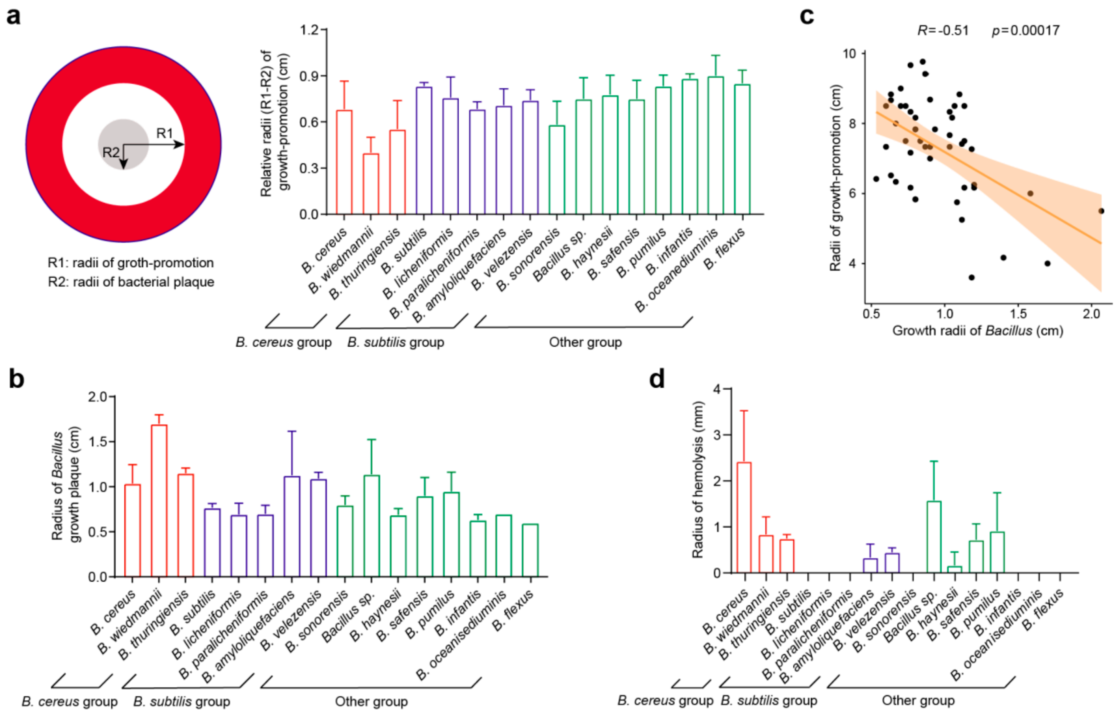

**Figure S1** ***Bacillus*** **promoting** ***A. paragallinarum*** **growth**.

**a-b** *Bacillus* facilitates the growth of *A. paragallinarum*. Diverse species of *Bacillus* cocultured with *A. paragallinarum* on blood agar at 37°C for 24 h. *A. paragallinarum* grew in proximity to the *Bacillus* microcolony but died far away from *Bacillus*. The growth radii of *A. paragallinarum* (a) and *Bacillus* (b) were recorded individually from the disc center to the edge of the microcolony.

**c** The negative correlation between growth promotion and *Bacillus* plaque. The error bands indicate the 95% confidence intervals.

**d** The hemolysis of *Bacillus*. *Bacillus* was cultivated overnight and diluted into $10^6$ cfu/mL, then 5 μL of bacterial suspensions were spotted on blood agar for 24 h culturing at 37°C. The transparent hemolytic zone was measured after cultivation. Mean values of radius are shown, with error bars indicating standard deviation (SD) for n⩾3.

**Table S2** Minimum inhibitory concentration for mono-cultures and co-cultures (1:1) of *Bacillus* and *A. paragallinarum*

| Antibiotics | Mono-culture (μg/mL) | | | Co-culture (μg/mL) | |
|---|---|---|---|---|---|
| | *A. paragallinarum* | *B. cereus* | *B. licheniformis* | *A. paragallinarum* | |
| | | CAU492 | YC 3-2 | CAU492 | YC 3-2 |
| Cefotaxime | <0.125 | 64 | 4 | 64 | 8 |
| Ofloxacin | 0.25 | <0.125 | <0.125 | 0.25 | 0.25 |
| Doxycycline | 8 | <0.125 | 0.5 | 16 | 16 |
| Gentamicin | <0.125 | 0.25 | <0.125 | 0.5 | 0.25 |
| Ampicillin | <0.125 | 64 | 0.25 | 64 | 0.25 |

NOTE: Red MIC values denote higher MIC values in the co-culture group compared to the *A. paragallinarum*-alone group.

**Table S3** Bacterial number of *A. paragallinarum* in mono-culture and co-culture

| Antibiotics | Mono-culture | Co-culture | |
|---|---|---|---|
| | *A. paragallinarum* X1-1S-1 (CFUs) | *A. paragallinarum* X1-1S-1 (CFUs) | |
| | | *B. cereus* CAU492 | *B. licheniformis* YC 3-2 |
| Cefotaxime | 0 | 1250 | 2250 |
| Ampicillin | 6 | ND | ND |

Note: *A. paragallinarum* count were determined in *A. paragallinarum*-alone group and the wells of the same concentration of antibiotic from the co-cultured wells. ND means the bacterial number is too much to count (>$10^6$ CFUs).

**Table S4** The presence of resistance genes and toxin genes in *A. paragallinarum* and *Bacillus* spp..

| Species | Resistant genes | Toxin genes |
|---|---|---|
| *A. paragallinarum* X1-1S-1 | *tet(B)* | *lpxC*, *manB*, *yhxB*, *gmhA*, *lpcA* |
| *B. cereus* CAU492 | *bcII, bcI*, *fosB, vanZF,* | *cytK, bas3190, nheC, nheA*, *inhA* |
| *B. licheniformis* YC 3-2 | *ermD, bcrA, bcrB, bcrC* | - |

Note: The presence of resistance genes and toxin genes were analyzed using the genomes of *A. paragallinarum* and *Bacillus* spp.. β-lactamases genes (*bcII* and *bcI*); hemolytic enterotoxin genes (*nheA* and *nheC*).
